# Cytokine-induced killer cells combined with temozolomide suppress glioblastoma progression in an orthotopic model and demonstrate clinical feasibility in recurrent glioblastoma

**DOI:** 10.3389/fneur.2026.1895857

**Published:** 2026-09-30

**Authors:** Young Kyu Park, Dong Won Yang, Kyun Woo Park, Gun He Nam, Choon-Soo Lee, Tae Whan Kim, Jong-Yeup Kim, Jong Gwon Choi

**Affiliations:** 1 Department of Oncology-Hematology, Konyang University Hospital, Daejeon, Republic of Korea; 2 Department of Biomedical Informatics, College of Medicine, Konyang University, Daejeon, Republic of Korea; 3 Department of Medical Oncology, Yuseong Sun General Hospital, Daejeon, Republic of Korea; 4 Product Development Division, GC Cell Corp., Yongin-si, Republic of Korea; 5 R&D M Unit, GC Cell Corp., Yongin-si, Republic of Korea

**Keywords:** adoptive cell therapy, cytokine-induced killer cells, glioblastoma, orthotopic model, recurrent glioblastoma, temozolomide

## Abstract

**Introduction:**

Glioblastoma (GBM) remains an aggressive primary brain tumor with limited therapeutic options after recurrence. Cytokine-induced killer (CIK) cells are ex vivo-expanded cytotoxic lymphocytes with T-cell and natural killer-like properties. We evaluated CIK-cell-based therapy using an orthotopic LN229-luciferase GBM model and a retrospective cohort of patients with recurrent GBM.

**Methods:**

Twelve independently manufactured patient-derived CIK-cell products were phenotypically characterized. In the orthotopic model, control, temozolomide (TMZ), and TMZ+CIK groups (n = 4 per group) were monitored by longitudinal bioluminescence imaging, with quantitative histological and immunohistochemical analyses. Clinical outcomes and safety were retrospectively evaluated in 12 heavily pretreated patients with recurrent GBM.

**Results:**

Expanded CIK-cell products showed a predominant effector-memory phenotype and high NKG2D and DNAM-1 expression. Longitudinal bioluminescence imaging demonstrated a significant treatment-by-time interaction among the three groups through Week 4 (*p* = 0.0054). At Week 4, both TMZ and TMZ+CIK showed significantly lower radiance than control, whereas TMZ and TMZ+CIK did not differ significantly. An exploratory comparison between TMZ and TMZ+CIK through Week 8 was not significant (*p* = 0.0663). Quantitative H&E analysis showed an overall group difference in tumor area fraction (*p* = 0.0376), whereas Granzyme B and CD56 staining did not differ significantly. In the clinical cohort, stable disease was the best recorded response in all evaluable patients; median progression-free survival was 10.5 months and median overall survival was 14.2 months. CIK-cell-based therapy was generally well tolerated.

**Discussion:**

These findings provide quantitative preclinical evidence of treatment-associated antitumor activity and support the clinical feasibility of CIK-cell-based therapy in recurrent GBM. However, the study does not establish a statistically significant incremental preclinical benefit of adding CIK cells to TMZ or clinical therapeutic efficacy. Prospective, adequately powered controlled studies are warranted.

## Introduction

Glioblastoma (GBM) is the most common and aggressive primary malignant brain tumor in adults and continues to be associated with poor clinical outcomes despite advances in multimodal treatment strategies (1). The current standard of care consists of maximal surgical resection followed by radiotherapy with concomitant and adjuvant temozolomide (TMZ). Nevertheless, disease recurrence is nearly universal, and therapeutic options for recurrent GBM remain limited. Although bevacizumab has been used in the recurrent setting, its survival benefit remains modest. Likewise, immune checkpoint inhibitors such as nivolumab have not demonstrated superior outcomes compared with bevacizumab in phase III trials, underscoring the challenges of immunotherapy in GBM (2).

Several immunotherapeutic strategies have been explored to overcome the highly immunosuppressive tumor microenvironment of GBM. Immune checkpoint blockade has shown limited efficacy, partly because GBM is characterized by relatively low tumor mutational burden, poor immune infiltration, marked intratumoral heterogeneity, and multiple immunosuppressive mechanisms within the central nervous system. Chimeric antigen receptor T-cell therapies have demonstrated tumor trafficking in early clinical studies but have generally failed to produce durable clinical responses in most patients with recurrent GBM (3). These observations highlight the need for alternative immune-based approaches capable of inducing broader and more flexible antitumor responses (4, 5).

Cytokine-induced killer (CIK) cell therapy is a form of adoptive immunotherapy generated by ex vivo stimulation of peripheral blood mononuclear cells with cytokines and anti-CD3 antibodies. The resulting cell population is heterogeneous and typically contains CD3^+^CD56^+^ NK-like T cells, CD3^+^CD56^−^ T cells, and minor NK-cell subsets (6). CIK cells can mediate both MHC-restricted and MHC-unrestricted tumor-cell killing, which may help overcome immune-evasion mechanisms that limit conventional T-cell-based therapies (7, 8).

Previous preclinical studies have suggested that CIK cells exert antitumor activity against glioblastoma models and that combination strategies with cytotoxic chemotherapy may enhance therapeutic activity (9, 10). Clinical studies have also evaluated CIK-cell-based immunotherapy incorporated into standard treatment strategies for newly diagnosed GBM. In a multicenter randomized phase III study, the addition of CIK cells to TMZ-based treatment improved progression-free survival and disease-control rates, although the overall survival benefit did not reach statistical significance (11, 12).

In the present study, we evaluated CIK-cell-based therapy in GBM using complementary preclinical and clinical approaches. First, we characterized expanded patient-derived CIK-cell products and evaluated treatment-associated antitumor activity of TMZ-containing regimens in an orthotopic LN229 glioblastoma model. Second, we described treatment patterns, clinical outcomes, and safety in a retrospective cohort of heavily pretreated patients with recurrent GBM who received CIK-cell-based therapy. These complementary analyses were intended to characterize preclinical activity and clinical feasibility rather than establish clinical efficacy.

## Methods

### CIK cell preparation and phenotypic analysis

CIK cells were generated from patient-derived peripheral blood mononuclear cells (PBMCs) at a GMP facility (GC Cell, Yongin-si, Republic of Korea) using the previously described manufacturing platform (12). Briefly, isolated mononuclear cells were suspended at an initial density of approximately 0.3–3 × 10^6^ cells/mL, activated using immobilized anti-CD3 monoclonal antibody, and expanded in medium containing recombinant human interleukin-2 (IL-2). Expanded cell products were harvested after approximately 12–21 days of culture. The final product was defined as the harvested, GMP-expanded PBMC-derived cell product after completion of the manufacturing process and applicable release assessments, including viability and microbiological safety testing such as sterility, mycoplasma, and endotoxin testing. The final product was not defined for this analysis by an arbitrary minimum proportion of CD3^+^CD56^+^ cells.

Phenotypic characterization was performed on 12 independently manufactured patient-derived final CIK-cell products, one product per patient (*n* = 12). No healthy-donor products were included. These 12 patients were distinct from the 12 patients included in the retrospective recurrent-GBM clinical cohort. Expanded products were analyzed by multiparameter flow cytometry using a BD LSRFortessa system (BD Biosciences, United States). Cells were harvested, washed with phosphate-buffered saline containing 1–2% fetal bovine serum or staining buffer, and stained with antibodies for 20–30 min at 4 °C in the dark before acquisition.

The following antibodies were used: anti-CD3-APC-Cy7 (BD Biosciences, Cat. No. 557832), anti-CD4-BV510 (BD Biosciences, Cat. No. 562970), anti-CD8-PE-Cy7 (BD Biosciences, Cat. No. 557746), anti-CD56-BV421 (BD Biosciences, Cat. No. 562751), anti-TCRαβ-BV786 (BD Biosciences, Cat. No. 563825), anti-TCRγδ-PerCP-Cy5.5 (BD Biosciences, Cat. No. 564157), anti-CD45RA-APC (BD Biosciences, Cat. No. 550855), anti-CCR7-PE (BioLegend, Cat. No. 353204), anti-NKG2D-BV605 (BD Biosciences, Cat. No. 743559), anti-DNAM-1-BV711 (BD Biosciences, Cat. No. 564796), anti-TIM-3-Alexa Fluor 647 (BD Biosciences, Cat. No. 565558), anti-PD-1-PE (BD Biosciences, Cat. No. 557946). Flow-cytometry data were analyzed using FlowJo software or equivalent cytometry-analysis software.

### Animal experiments and orthotopic GBM model

All animal procedures were approved by the Institutional Animal Care and Use Committee of WOOJUNG BIO (IACUC No. IACUC2003-033) and were conducted in accordance with the Guide for the Care and Use of Laboratory Animals. All efforts were made to minimize animal pain, suffering, and distress.

Female BALB/cAJcl-nu/nu mice, 6 weeks of age and weighing 18.0–20.0 g, were purchased from Saeronbio (Republic of Korea) and sourced from CLEA Japan, Inc. (Tokyo, Japan). Mice were housed under controlled conditions at 22 ± 2 °C and 50 ± 15% relative humidity under a 12-h light/dark cycle, with ad libitum access to food and water. Animals were housed at five mice per cage and acclimatized to the facility for at least 7 days before tumor implantation.

LN229 was selected as a complementary human GBM model to the U-87MG model used in a previous preclinical CIK-cell study (9). LN229 is a tumorigenic human glioblastoma cell line with reported molecular alterations including a TP53 P98L mutation, wild-type PTEN, and homozygous deletions involving p16/CDKN2A and p14ARF (13, 14). Comparative orthotopic studies have shown that LN229 tumors exhibit a more diffuse and infiltrative growth pattern, including invasion into surrounding brain parenchyma, whereas U-87MG tumors tend to form more circumscribed tumor masses (15). Luciferase-expressing LN229 cells have also been used for non-invasive longitudinal monitoring of intracranial tumor growth and treatment response (16). The present model was not intended to represent a specific model of recurrent or TMZ-resistant GBM; rather, it was used to model infiltrative intracranial GBM growth and permit serial assessment of treatment response.

Orthotopic GBM xenografts were established using luciferase-expressing LN229 cells. For stereotactic tumor implantation, mice were anesthetized by intramuscular injection of a tiletamine-zolazepam and xylazine mixture. Zoletil 50 (Virbac, Carros, France), containing 125 mg each of tiletamine and zolazepam per vial, was reconstituted with 5 mL of the supplied diluent. The reconstituted Zoletil solution (5.0 mL) was then mixed with 1.66 mL of xylazine hydrochloride (Rompun; Bayer, Republic of Korea) to prepare an approximately 3:1 (v/v) mixture. The anesthetic mixture was administered intramuscularly at 1.0 mL/kg body weight, corresponding to approximately 18.8 mg/kg each of tiletamine and zolazepam and 5.0 mg/kg xylazine base (approximately 5.8 mg/kg xylazine hydrochloride).

Mice were positioned in a stereotaxic apparatus (Harvard Apparatus, Holliston, MA, United States). A burr hole was made at coordinates 1 mm anterior and 2 mm lateral to the bregma. LN229-luciferase cells (1 × 10^5^ cells suspended in 3 μL Hank’s balanced salt solution) were slowly injected over 2 min at a depth of 3.5 mm using a Hamilton syringe.

Tumor establishment and progression were monitored longitudinally by *in vivo* bioluminescence imaging (BLI) using an IVIS Lumina LT Series III imaging system (PerkinElmer, United States). For each imaging session, D-luciferin was administered intraperitoneally at 150 mg/kg in a volume of 200 μL per mouse. Mice were anesthetized with 3.5% isoflurane in an induction chamber, and anesthesia was maintained with 3.5% isoflurane during image acquisition. Images were acquired using fixed settings: 60-s exposure time, binning of 2, f/stop of 1.2, field of view of 24, blocked excitation filter, open emission filter, and no auto-exposure. For quantitative analysis, a whole-body region of interest extending approximately from the nose to the base of the tail was manually adjusted according to the body size of each animal. Radiance was expressed as photons/s/cm^2^/sr. For consistent visualization, the aggregate display color scale was fixed at 1 × 10^5^ to 1 × 10^7^ across groups and time points; quantitative analyses were based on the numerical ROI radiance values and were independent of the displayed color scale. Absolute ROI radiance values were analyzed without baseline normalization or baseline correction, and no numerical BLI observations were excluded as statistical outliers.

Following confirmation of intracranial tumor formation, mice were randomly assigned to the control, TMZ, or TMZ + CIK group (*n* = 4 per group). No formal *a priori* power calculation was performed for the original animal study. The treatment-initiation time point was designated as Day 0. TMZ was administered orally at 5 mg/kg once daily for 7 consecutive days beginning on Day 0. CIK cells were administered intravenously at 1 × 10^7^ cells per mouse once weekly for a total of eight administrations on Days 0, 7, 14, 21, 28, 35, 42, and 49. BLI was performed at approximately weekly intervals through Week 8. Body weight and clinical signs were recorded twice weekly. The combination strategy was supported by prior orthotopic GBM studies of TMZ with CIK cells (9), while a separate orthotopic patient-derived GBM study demonstrated biological activity of TMZ at the 5 mg/kg dose level (17); the TMZ schedules in those studies were not identical to the regimen used here.

At the predefined experimental endpoint, or earlier when humane endpoint criteria were met, mice were euthanized using medical-grade carbon dioxide. Mice were placed in the euthanasia chamber before carbon dioxide was introduced; the chamber was not prefilled with carbon dioxide. Carbon dioxide was delivered from a medical-grade gas cylinder at approximately 20 L/min. Respiratory and cardiac arrest were confirmed before tissue collection. When necessary, cervical dislocation was performed as a secondary physical method to ensure death before collection of brain tissues for histological and immunohistochemical analyses.

### Histological and immunohistochemical analysis of mouse brain tissues

At the designated endpoint, brain tissues were collected for histological and immunohistochemical evaluation. Whole brains were fixed in 10% neutral-buffered formalin, embedded in paraffin, and sectioned for histological analysis. H&E-stained sections were used to assess histologically identifiable tumor involvement and brain architecture.

For the additional quantitative histomorphometric analysis performed during revision, one available low-magnification H&E-stained coronal brain section from each independent animal was analyzed for the control (*n* = 3), TMZ (*n* = 3), and TMZ + CIK (*n* = 2) groups. Images were assigned randomized identifiers and analyzed before treatment-group unblinding. Tumor involvement was quantified as the percentage of histologically identifiable tumor area relative to the total evaluable brain-tissue area using ImageJ-based histomorphometry. Because a single available section per animal was analyzed, this endpoint represents tumor area fraction within the analyzed section and not a stereologic estimate of total intracranial tumor volume.

For immunohistochemical analysis, paraffin-embedded sections were deparaffinized and rehydrated, followed by antigen retrieval using an appropriate antigen retrieval buffer. Endogenous peroxidase activity was blocked using hydrogen peroxide, and nonspecific binding was blocked with a suitable blocking solution. Sections were then incubated with primary antibodies against granzyme B (Abcam, Cat. No. ab255598) and CD56 (Cell Signaling, Cat. No. 99746S). After primary antibody incubation, sections were incubated with an appropriate horseradish peroxidase-conjugated secondary antibody, developed using a chromogenic substrate such as 3,3′-diaminobenzidine (DAB), and counterstained with hematoxylin.

Available CD56- and Granzyme B-stained sections from independent animals in the control (*n* = 3), TMZ (*n* = 3), and TMZ + CIK (*n* = 2) groups were quantitatively analyzed using H-DAB color deconvolution, standardized ROI criteria, and a fixed marker-specific threshold applied consistently across treatment groups. The quantitative endpoint was DAB-positive area fraction. Negative-control sections were not included in the original IHC staining series and were unavailable for the present analysis. The administered CIK cells were not directly labeled or tracked, and species-specific validation sufficient to establish the cellular origin of CD56-positive staining was not performed.

### Clinical study design and patient eligibility

A multicenter, retrospective observational study was conducted at Konyang University Hospital (IRB No. KYUH 2023-06-044-002) and Yuseong Sun General Hospital (IRB No. YSSH 202310-001). Medical records of patients with GBM who received CIK-cell immunotherapy between January 2015 and December 2023 were reviewed.

The inclusion criteria were as follows: histologically confirmed GBM; receipt of at least three cycles of CIK-cell therapy; and prior treatment with standard GBM therapies, including surgery, radiotherapy, and/or chemotherapy. Patients with incomplete follow-up information or insufficient medical records for outcome assessment were excluded. The requirement for informed consent was waived by the institutional review boards because of the retrospective nature of the study. The study was conducted in accordance with the principles of the Declaration of Helsinki and applicable institutional guidelines.

CIK cells were administered intravenously at 2-week intervals. Documented concomitant regimens included TMZ, nivolumab, or bevacizumab-based therapy. TMZ was administered at 75 mg/m^2^ daily when used in combination. Nivolumab was administered at 240 mg every 2 weeks for 8 cycles, followed by 480 mg every 4 weeks. Bevacizumab was administered at 10 mg/kg every 2 weeks.

Molecular characteristics were obtained from institutional pathology records. IDH status was assessed by immunohistochemistry, MGMT promoter methylation status by pyrosequencing, and EGFR amplification by fluorescence *in situ* hybridization (FISH).

Tumor response was assessed according to RECIST version 1.1, with brain magnetic resonance imaging performed at approximately 8-week intervals. Stable disease was defined as neither sufficient tumor shrinkage to qualify for partial response nor sufficient increase in tumor burden to qualify for progressive disease according to RECIST 1.1. No blinded independent central review was performed; response and survival outcomes were derived from routine clinical records and imaging. The exact number of recurrences was not consistently documented as a separate variable, and treatment line was therefore reported without using it as a surrogate for recurrence number. Adverse events were graded according to the Common Terminology Criteria for Adverse Events, version 4.03.

### Statistical analysis

For the paired comparison of CD3^+^CD56^−^ T-cell and CD3^+^CD56^+^ CIK-cell proportions within the same independently manufactured CIK-cell products, a two-tailed Wilcoxon matched-pairs signed-rank test was used.

For longitudinal BLI analysis, radiance values were log10-transformed because of their wide dynamic range. The primary three-group inferential analysis was restricted to baseline through Week 4, during which complete repeated measurements were available for all animals in the control, TMZ, and TMZ + CIK groups (*n* = 4 per group). Log10-transformed radiance values were analyzed using two-way repeated-measures ANOVA with treatment group and time as factors. Sphericity was not assumed, and the Geisser–Greenhouse correction was applied. The treatment-by-time interaction was used to assess differences in longitudinal BLI trajectories, and Tukey-adjusted comparisons were performed between treatment groups within individual time points. One mouse in the control group died from tumor progression after Week 4; subsequent measurements were unavailable and no post-death values were imputed. Because complete longitudinal measurements were available for the TMZ and TMZ + CIK groups through Week 8, an additional exploratory full-period repeated-measures analysis was performed for these two groups using two-way repeated-measures ANOVA with Geisser–Greenhouse correction and Šídák-adjusted between-group comparisons at individual time points.

For quantitative histological and immunohistochemical analyses, tumor area fraction measured on low-magnification H&E-stained sections and DAB-positive area fractions for Granzyme B and CD56 were compared among treatment groups using the Kruskal–Wallis test. Given the limited number of available sections, these analyses were considered exploratory.

Progression-free survival (PFS) and overall survival (OS) in the retrospective clinical cohort were estimated using the Kaplan–Meier method and are reported with 95% confidence intervals (CIs). PFS was defined as the time from initiation of CIK-cell-based therapy to documented disease progression or death from any cause, whichever occurred first. OS was defined as the time from initiation of CIK-cell-based therapy to death from any cause. For PFS, patients without documented progression or death were censored at the date of last follow-up; for OS, patients who were alive were censored at the date of last follow-up. Survival curves were compared using the log-rank test when applicable.

Because of the small sample size, retrospective design, and non-randomized treatment allocation in the clinical cohort, subgroup analyses were considered exploratory and descriptive. No adjustment for multiplicity was performed for these clinical subgroup analyses. All statistical tests were two-sided, and *p* values < 0.05 were considered statistically significant. Statistical analyses were performed using SPSS version 27.0 (IBM Corp., Armonk, NY, United States) and GraphPad Prism version 11.0.2 (GraphPad Software, Boston, MA, United States).

## Results

### Phenotypic characterization of expanded patient-derived CIK cells

The 12 independently manufactured patient-derived CIK-cell products showed a heterogeneous immune-cell composition consisting mainly of CD3^+^CD56^−^ T cells and CD3^+^CD56^+^ CIK cells, with a minor CD3^−^CD56^+^ NK-cell population. CD3^+^CD56^−^ T cells accounted for 72.8% and CD3^+^CD56^+^ CIK cells for 26.1% of the expanded products. A two-tailed Wilcoxon matched-pairs signed-rank test demonstrated a significantly higher proportion of CD3^+^CD56^−^ T cells than CD3^+^CD56^+^ CIK cells (exact *p* = 0.0005; *n* = 12). CD3^−^CD56^+^ NK cells accounted for 0.8%. Among T-cell receptor-defined subsets, αβ T cells and γδ T cells represented 88.5 and 8.4% of the population, respectively (Figure 1A).

**Figure 1:**
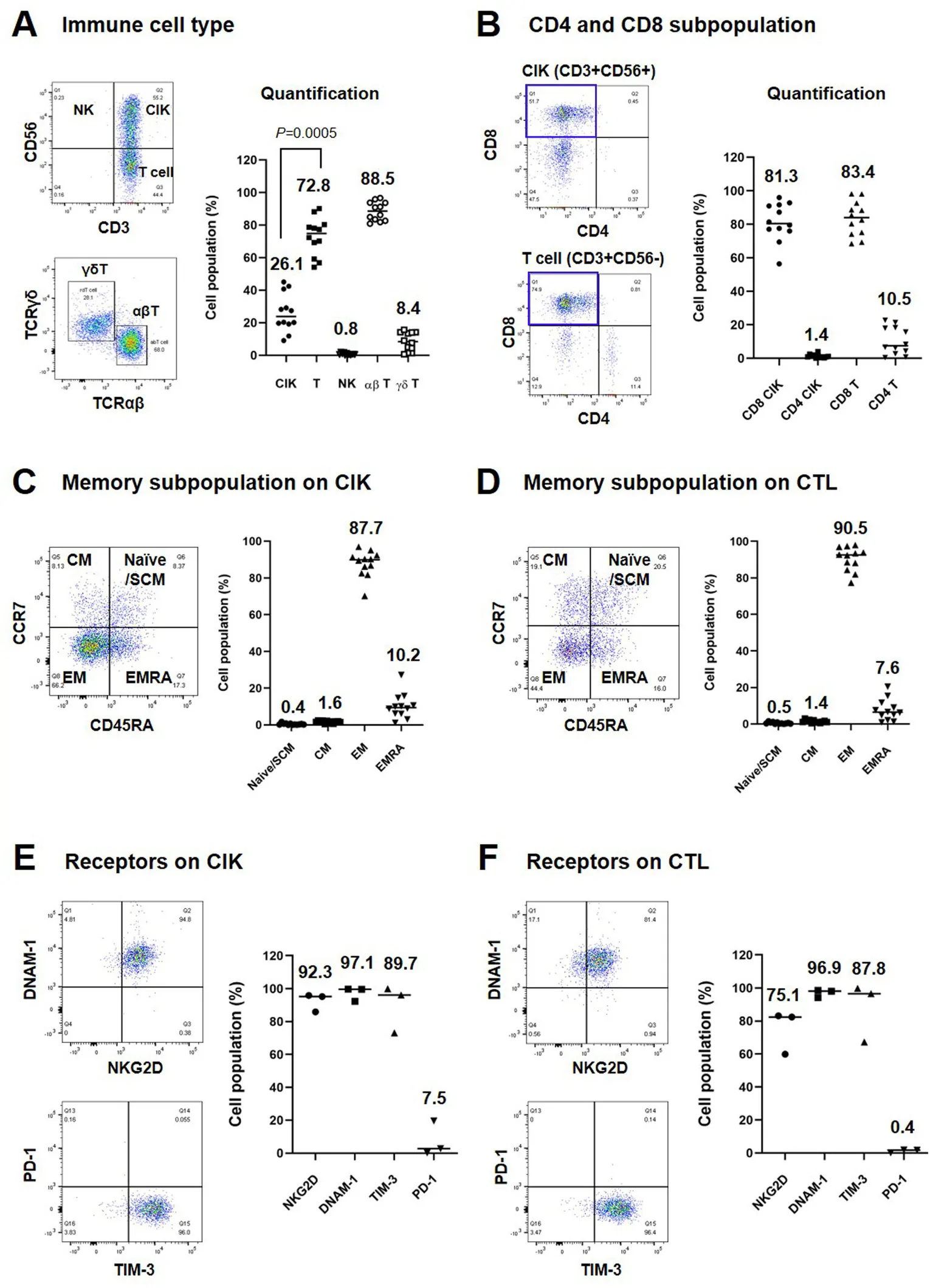
Phenotypic characterization of patient-derived CIK-cell products by flow cytometry. Peripheral blood mononuclear cell-derived CIK-cell products were analyzed by flow cytometry after *ex vivo* expansion. Data were obtained from 12 independently manufactured patient-derived CIK-cell products, one from each patient; these patients were distinct from the 12 patients included in the retrospective recurrent-GBM clinical cohort. **(A)** Representative flow-cytometry plots and quantification of major immune-cell populations within the expanded products, including CIK cells (CD3^+^CD56^+^), CD3^+^CD56^−^ T cells, natural killer (NK) cells (CD3^−^CD56^+^), αβ T cells, and γδ T cells. CD3^+^CD56^−^ T cells accounted for 72.8% and CD3^+^CD56^+^ CIK cells for 26.1% of the expanded products. A two-tailed Wilcoxon matched-pairs signed-rank test demonstrated a significant difference between these paired populations (exact *p* = 0.0005; *n* = 12). **(B)** Representative flow-cytometry plots and quantification of CD8^+^ and CD4^+^ subsets within CD3^+^CD56^+^ CIK cells and CD3^+^CD56^−^ T cells (*n* = 12). **(C)** Representative flow-cytometry plot and quantification of memory subsets in CIK cells based on CD45RA and CCR7 expression. Subsets were defined as naïve/stem cell memory (naïve/SCM; CD45RA^+^CCR7^+^), central memory (CM; CD45RA^−^CCR7^+^), effector memory (EM; CD45RA^−^CCR7^−^), and terminally differentiated effector memory re-expressing CD45RA (EMRA; CD45RA^+^CCR7^−^). **(D)** Representative flow-cytometry plot and quantification of memory subsets in CD3^+^CD56^−^ T cells using the same CD45RA/CCR7-based classification. EM cells were the predominant subset in both populations (*n* = 12). **(E)** Representative flow-cytometry plots and quantification of NKG2D, DNAM-1, TIM-3, and PD-1 expression on CIK cells. **(F)** Representative flow-cytometry plots and quantification of NKG2D, DNAM-1, TIM-3, and PD-1 expression on CD3^+^CD56^−^ T cells. Data are presented as individual values with mean ± SD.

Both the CD3^+^CD56^+^ CIK-cell and CD3^+^CD56^−^ T-cell compartments were predominantly CD8-positive. CD8^+^ cells accounted for 81.3% of the CIK-cell fraction and 83.4% of the CD3^+^CD56^−^ T-cell fraction, whereas CD4^+^ cells represented 1.4 and 10.5%, respectively (Figure 1B).

Memory-subset analysis showed a predominant effector-memory phenotype. Among CIK cells, effector-memory cells represented 87.7%, followed by EMRA cells at 10.2%, central-memory cells at 1.6%, and naïve/stem-cell-memory cells at 0.4% (Figure 1C). A similar distribution was observed in the CD3^+^CD56^−^ T-cell compartment, where effector-memory cells represented 90.5%, followed by EMRA cells at 7.6%, central-memory cells at 1.4%, and naïve/stem-cell-memory cells at 0.5% (Figure 1D).

NKG2D and DNAM-1 were expressed on 92.3 and 97.1% of CD3^+^CD56^+^ CIK cells, respectively. TIM-3 and PD-1 were expressed on 89.7 and 7.5% of CIK cells, respectively (Figure 1E). In the CD3^+^CD56^−^ T-cell compartment, NKG2D, DNAM-1, TIM-3, and PD-1 expression was 75.1, 96.9, 87.8, and 0.4%, respectively (Figure 1F). These receptor data were interpreted as phenotypic observations; no direct functional cytotoxicity or exhaustion assays were performed.

### Treatment-associated antitumor activity in an orthotopic LN229 glioblastoma model

Following confirmation of intracranial tumor establishment, mice were assigned to the control, TMZ, or TMZ + CIK groups (*n* = 4 per group), and tumor burden was monitored longitudinally by weekly BLI (Figures 2A–C). For the primary analysis from baseline through Week 4, two-way repeated-measures ANOVA of log10-transformed BLI radiance demonstrated a significant treatment-by-time interaction (*F* (3.916, 17.62) = 5.368, *p* = 0.0054). At Week 4, Tukey-adjusted comparisons showed significantly lower BLI radiance in the TMZ group versus control (mean difference, 1.807 log10 radiance units; 95% CI, 0.2792–3.335; adjusted *p* = 0.0279) and in the TMZ + CIK group versus control (mean difference, 1.718; 95% CI, 0.6425–2.794; adjusted *p* = 0.0069). No significant difference was observed between TMZ and TMZ + CIK at Week 4 (mean difference, −0.08879; 95% CI, −1.635 to 1.457; adjusted *p* = 0.9817). One mouse in the control group (mouse No. 3) died from tumor progression after Week 4, and no post-death values were imputed. An exploratory full-period analysis restricted to the TMZ and TMZ + CIK groups through Week 8 showed numerical divergence at later time points but no statistically significant treatment-by-time interaction (*F* (1.999, 12.00) = 3.431, *p* = 0.0663); the Week 8 comparison was also not significant after Šídák adjustment (mean difference, 1.435; 95% CI, −1.600 to 4.470; adjusted *p* = 0.5753) (Figure 2D).

**Figure 2:**
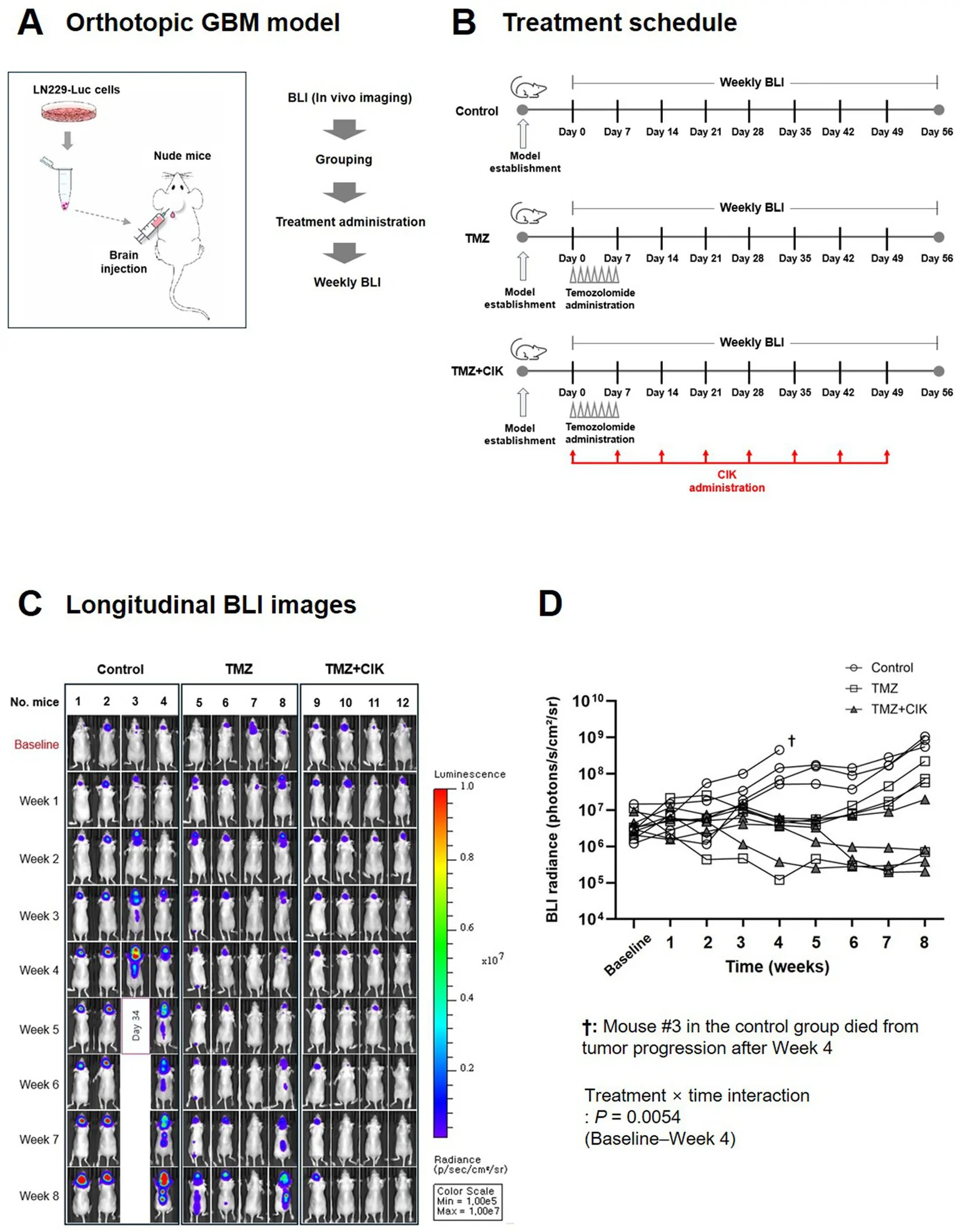
Experimental design and longitudinal antitumor activity of TMZ and TMZ plus CIK cells in an orthotopic LN229 glioblastoma model. **(A)** Schematic overview of the orthotopic LN229-luciferase GBM model and experimental workflow. **(B)** Treatment and weekly BLI schedule for the control, TMZ, and TMZ + CIK groups (*n* = 4 per group). **(C)** Longitudinal BLI images of individual mice showing intracranial tumor progression during the 8-week observation period. **(D)** Quantitative longitudinal BLI radiance in individual mice. Analysis of log10-transformed radiance from baseline through Week 4 demonstrated a significant treatment-by-time interaction (*p* = 0.0054). At Week 4, BLI radiance was significantly lower in the TMZ (adjusted *p* = 0.0279) and TMZ + CIK (adjusted *p* = 0.0069) groups than in the control group, whereas TMZ and TMZ + CIK did not differ significantly (adjusted *p* = 0.9817). An exploratory full-period comparison between TMZ and TMZ + CIK did not reach statistical significance (treatment-by-time interaction, *p* = 0.0663). Mouse No. 3 in the control group died from tumor progression after Week 4; no post-death values were imputed.

Quantitative histomorphometry of low-magnification H&E-stained sections showed a median tumor area fraction of 9.46% (range, 5.03–38.82%; *n* = 3) in the control group, whereas no histologically identifiable tumor area was detected in the analyzed sections from the TMZ group (0%; *n* = 3) or the TMZ + CIK group (0%; *n* = 2). An exploratory Kruskal–Wallis test demonstrated an overall group difference (*p* = 0.0376) (Figure 3A). These measurements reflect tumor involvement within the analyzed sections and not total intracranial tumor volume.

**Figure 3:**
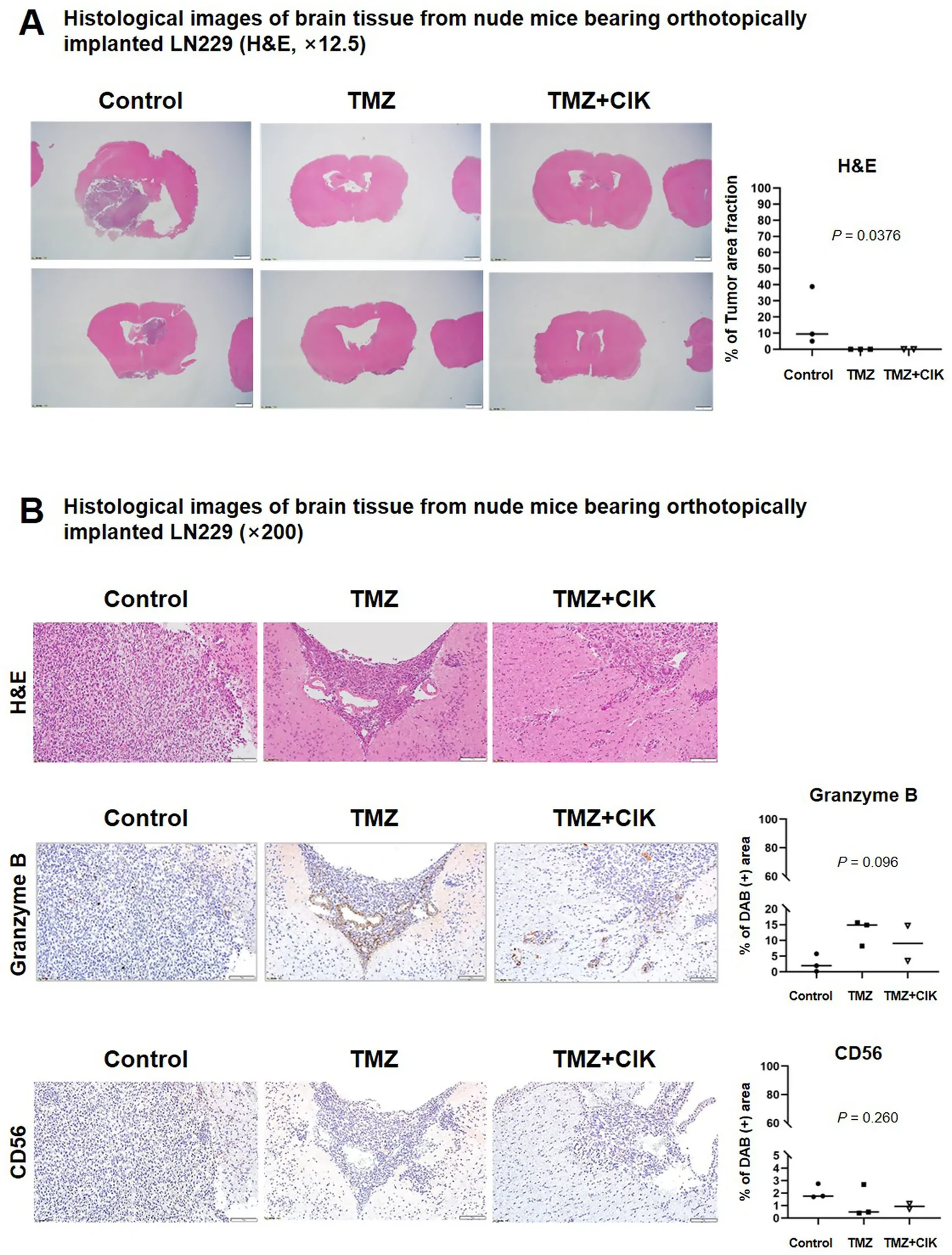
Quantitative histological and immunohistochemical analyses of brain tissues in the orthotopic LN229 glioblastoma model. **(A)** Representative low-magnification H&E-stained coronal brain sections and quantitative tumor area fraction. An overall group difference was observed (Kruskal–Wallis *p* = 0.0376). **(B)** Representative high-magnification H&E, Granzyme B, and CD56 staining with quantitative DAB-positive area analyses. Each analyzed image was obtained from a different mouse. Group differences were not significant for Granzyme B (*p* = 0.096) or CD56 (*p* = 0.260; Kruskal–Wallis tests).

Quantitative DAB-positive area analysis showed Granzyme B-positive area fractions of 2.63 ± 2.84%, 12.91 ± 4.10%, and 9.09 ± 7.92% in the control, TMZ, and TMZ + CIK groups, respectively; the overall group difference was not statistically significant (Kruskal–Wallis *p* = 0.096). CD56-positive area fractions were 2.07 ± 0.58%, 1.20 ± 1.30%, and 0.93 ± 0.30%, respectively, with no significant overall difference (Kruskal–Wallis *p* = 0.260) (Figure 3B). Because administered CIK cells were not directly labeled or tracked, species-specific validation was not performed, and negative-control sections were unavailable, CD56 staining was not interpreted as evidence of CIK-cell infiltration, and Granzyme B staining was not interpreted as direct evidence of CIK-specific cytotoxic activity.

### Clinical characteristics and real-world outcomes in patients with recurrent GBM

Twelve heavily pretreated patients with recurrent GBM received CIK-cell-based therapy. Median age at first CIK-cell administration was 51.0 years (range, 16–67), and 5 patients (41.7%) were male and 7 (58.3%) were female. Four patients (33.3%) had an ECOG performance status of 0–1 and 8 (66.7%) had a status of ≥2. Patients had received a median of 3 prior treatment lines (range, 2–5) and a median of 4.5 CIK-cell administrations (range, 3–20) (Table 1).

**Table 1:** Baseline and treatment characteristics of patients.

Variable	CIK-cell immunotherapy group (*n* = 12)
Median age at first CIK-cell administration, years (range)	51.0 (16–67)
Sex, *n* (%)	
Male	5 (41.7%)
Female	7 (58.3%)
Median number of CIK-cell administrations, (range)	4.5 (3–20)
ECOG performance status, *n* (%)	
PS 0–1	4 (33.3%)
PS ≥ 2	8 (66.7%)
Molecular characteristics, *n* (%)	
*IDH status (IHC)*	
Wild-type	8 (66.7%)
Mutant	2 (16.7%)
Not evaluated	2 (16.7%)
*MGMT promoter methylation status (pyrosequencing)*	
Methylated	2 (16.7%)
Unmethylated	10 (83.3%)
*EGFR amplification (FISH)*	
Amplified	2 (16.7%)
Non-amplified	10 (83.3%)
Median lines of standard therapy (range)	3 (2–5)
Prior treatment, *n* (%)	
Temozolomide with Radiotherapy	12 (100.0)
Irinotecan	5 (41.6)
Radiotherapy	4 (33.3)
Combination treatment, *n* (%)	
Bevacizumab	4 (33.3)
Bevacizumab plus irinotecan	2 (16.7)
Nivolumab	2 (16.7)
Ipilimumab plus nivolumab	1 (8.3)
Temozolomide	3 (25.0)
Tumor size, *n* (%)	
≤4 cm	8 (66.7)
>4 cm	4 (33.3)

CIK, cytokine-induced killer; ECOG, Eastern Cooperative Oncology Group; PS, performance status; IDH, isocitrate dehydrogenase; IHC, immunohistochemistry; MGMT, O^6^-methylguanine-DNA methyltransferase; EGFR, epidermal growth factor receptor; FISH, fluorescence in situ hybridization.

Molecular characterization showed IDH-wild-type status in 8 patients (66.7%) and IDH-mutant status in 2 (16.7%), while IDH status was not evaluated in 2 patients (16.7%). MGMT promoter methylation assessed by pyrosequencing was identified in 2 patients (16.7%), whereas 10 (83.3%) were classified as unmethylated. EGFR amplification assessed by FISH was detected in 2 patients (16.7%), while 10 (83.3%) were non-amplified (Table 1).

The swimmer plot summarizes the verified concomitant regimens administered with CIK-cell therapy and the individual clinical courses (Figure 4). No complete or partial responses were recorded. Stable disease, defined according to RECIST 1.1, was the best recorded response in all evaluable patients, with brain MRI performed at approximately 8-week intervals. Individual demographic, treatment-exposure, and clinical outcome data are summarized in Table 2.

**Figure 4:**
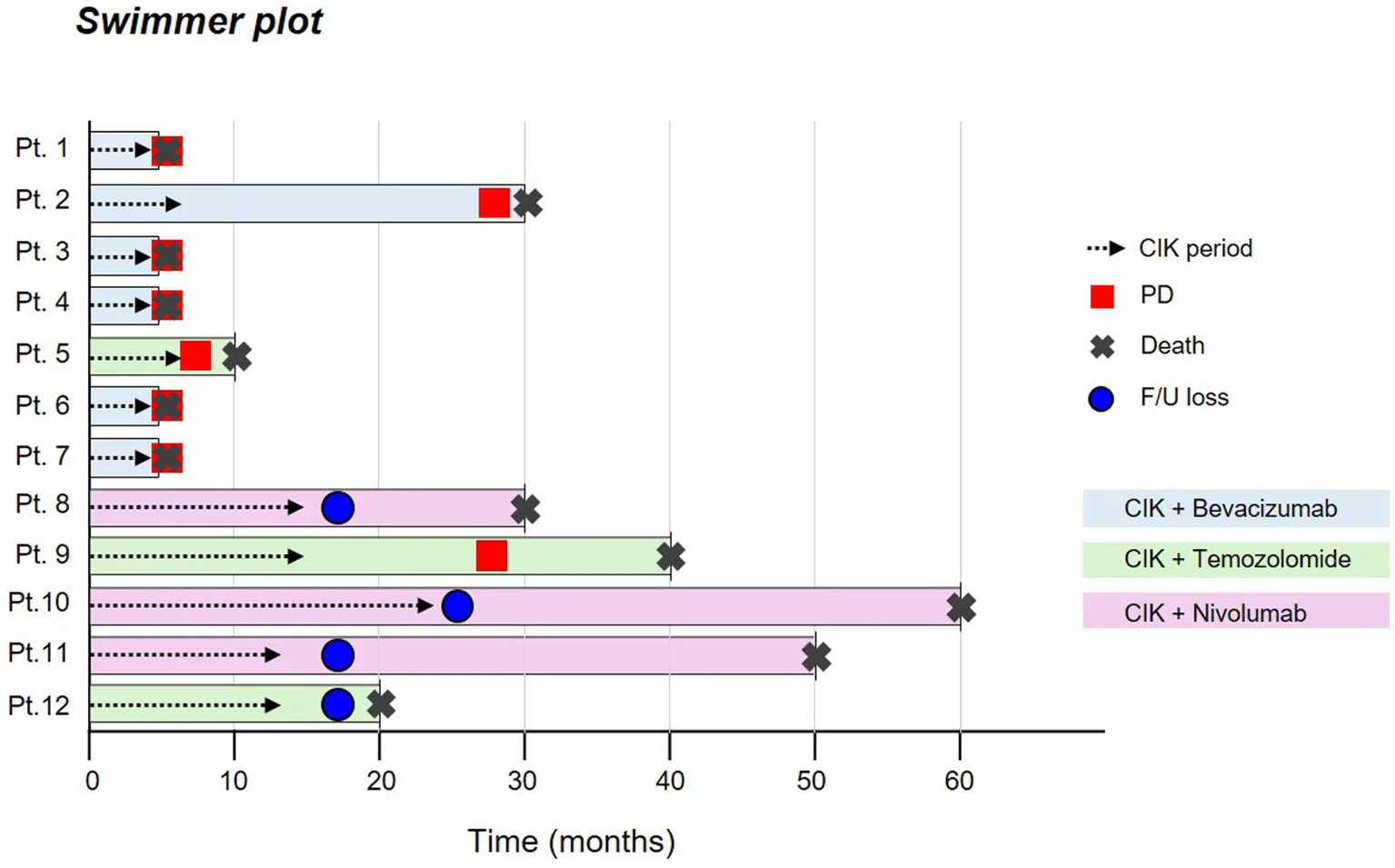
Individual treatment courses and clinical outcomes in patients with recurrent glioblastoma receiving CIK-cell-based therapy. Swimmer plot showing individual clinical courses in the retrospective recurrent GBM cohort (*n* = 12). Each horizontal bar represents one patient and indicates the duration of clinical follow-up. Dotted arrows indicate the CIK-cell treatment period. Red squares indicate progressive disease (PD), gray crosses indicate death, and blue circles indicate loss to follow-up. Background shading indicates the verified concomitant regimen administered with CIK-cell therapy, including CIK plus Bevacizumab, CIK plus Temozolomide, and CIK plus Nivolumab.

**Table 2:** Individual patient characteristics and clinical outcomes (*n* = 12).

Patient	Age (years)	Sex	IDH status	CIK cycles	Treat line	Best response	PFS (months)	OS (months)
Pt. 1	16	Male	IDH-wildtype	3 cycles	3rd	SD	2.3	2.3
Pt. 2	46	Female	Not evaluated	6 cycles	2nd	SD	28.4	28.8
Pt. 3	18	Male	IDH-wildtype	3 cycles	2nd	SD	6.9	6.9
Pt. 4	60	Female	Not evaluated	4 cycles	2nd	SD	4.8	4.8
Pt. 5	58	Female	IDH-mutant	5 cycles	5th	SD	2.3	8.4
Pt. 6	16	Male	IDH-wildtype	3 cycles	2nd	SD	2.2	3.1
Pt. 7	67	Female	IDH-wildtype	3 cycles	2nd	SD	5.4	6.2
Pt. 8	56	Female	IDH-mutant	5 cycles	4th	SD	17.2	25.1
Pt. 9	24	Male	IDH-wildtype	9 cycles	3rd	SD	28.6	36.4
Pt. 10	60	Female	IDH-wildtype	20 cycles	3rd	SD	14.1	56.4
Pt. 11	57	Male	IDH-wildtype	3 cycles	3rd	SD	24.0	48.6
Pt. 12	41	Female	IDH-wildtype	8 cycles	3rd	SD	16.6	20.1

Age was calculated at the date of first CIK-cell administration. The exact number of recurrences was not consistently documented as a separate variable; treatment line is reported but was not used as a surrogate for recurrence number. Tumor response was assessed according to RECIST version 1.1 with brain MRI performed at approximately 8-week intervals. CIK, cytokine-induced killer; IDH, isocitrate dehydrogenase; SD, stable disease; PFS, progression-free survival; OS, overall survival.

Kaplan–Meier analysis showed a median PFS of 10.5 months (95% CI, 4.8– not available) and a median OS of 14.2 months (95% CI, 6.2– not available) (Figures 5A,B). These values represent descriptive outcomes of the retrospective cohort and cannot establish a causal survival benefit attributable to CIK-cell therapy in the absence of a comparable control group.

**Figure 5:**
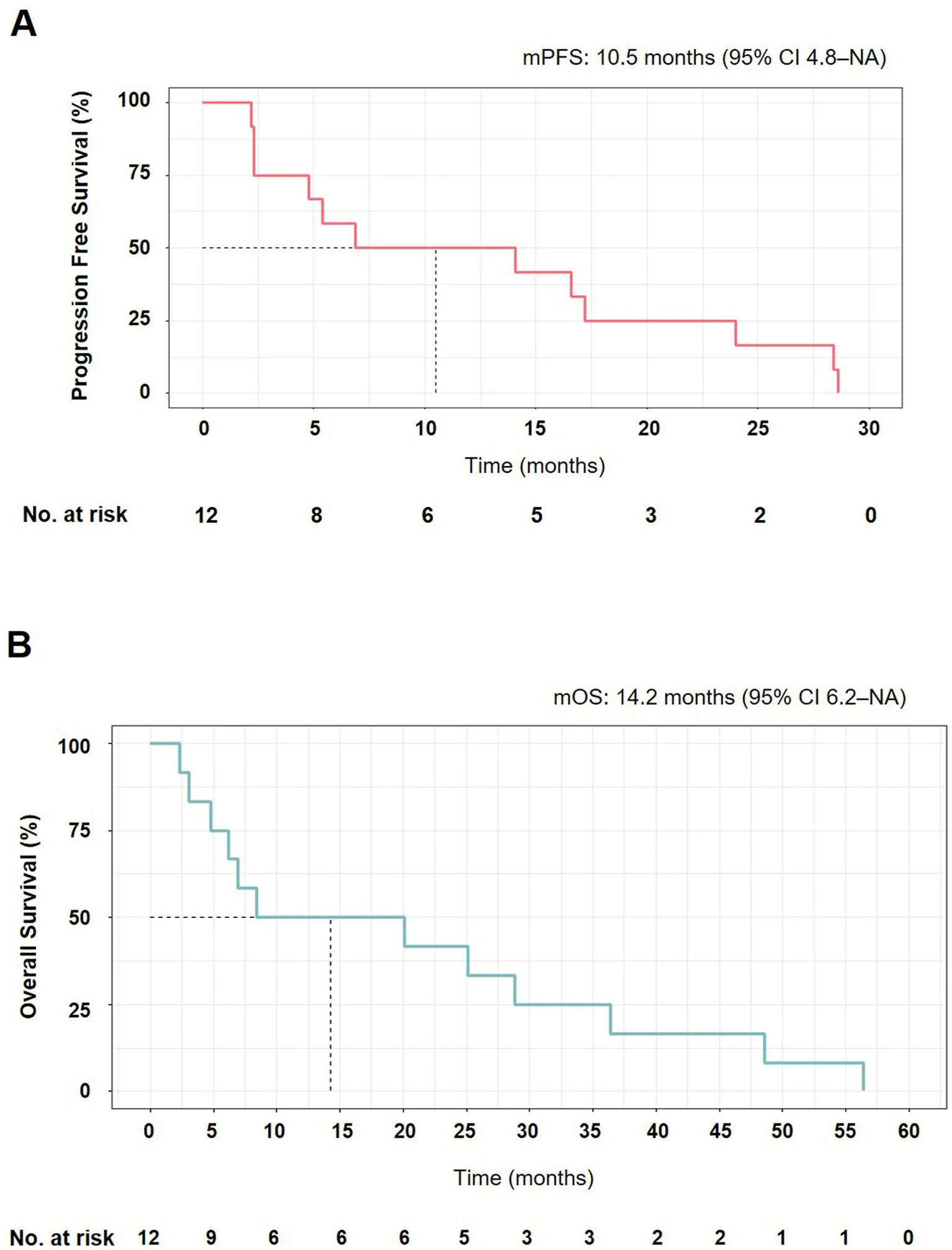
Kaplan–Meier estimates of progression-free survival and overall survival in the overall recurrent glioblastoma cohort. Kaplan–Meier curves showing progression-free survival (PFS) and overall survival (OS) in patients with recurrent glioblastoma receiving CIK-cell-based therapy in the retrospective cohort (*n* = 12). **(A)** PFS in the overall cohort. Median PFS was 10.5 months (95% confidence interval [CI], 4.8–NA). **(B)** OS in the overall cohort. Median OS was 14.2 months (95% CI, 6.2–NA). Numbers at risk are shown below each panel, and censoring marks are shown on the curves where applicable. For PFS, patients without documented progression or death were censored at the date of last follow-up; for OS, patients who were alive were censored at the date of last follow-up. NA, not available.

Exploratory subgroup analyses were performed according to concomitant regimen. Patients receiving CIK plus bevacizumab (*n* = 6) had a median PFS of 5.1 months and median OS of 5.5 months, whereas patients receiving CIK plus nivolumab (*n* = 3) had a median PFS of 17.2 months and median OS of 48.6 months (Figures 6A,B). Given the very small subgroup sizes and non-randomized treatment allocation, these findings are descriptive and hypothesis-generating and should not be interpreted as evidence of comparative treatment efficacy.

**Figure 6:**
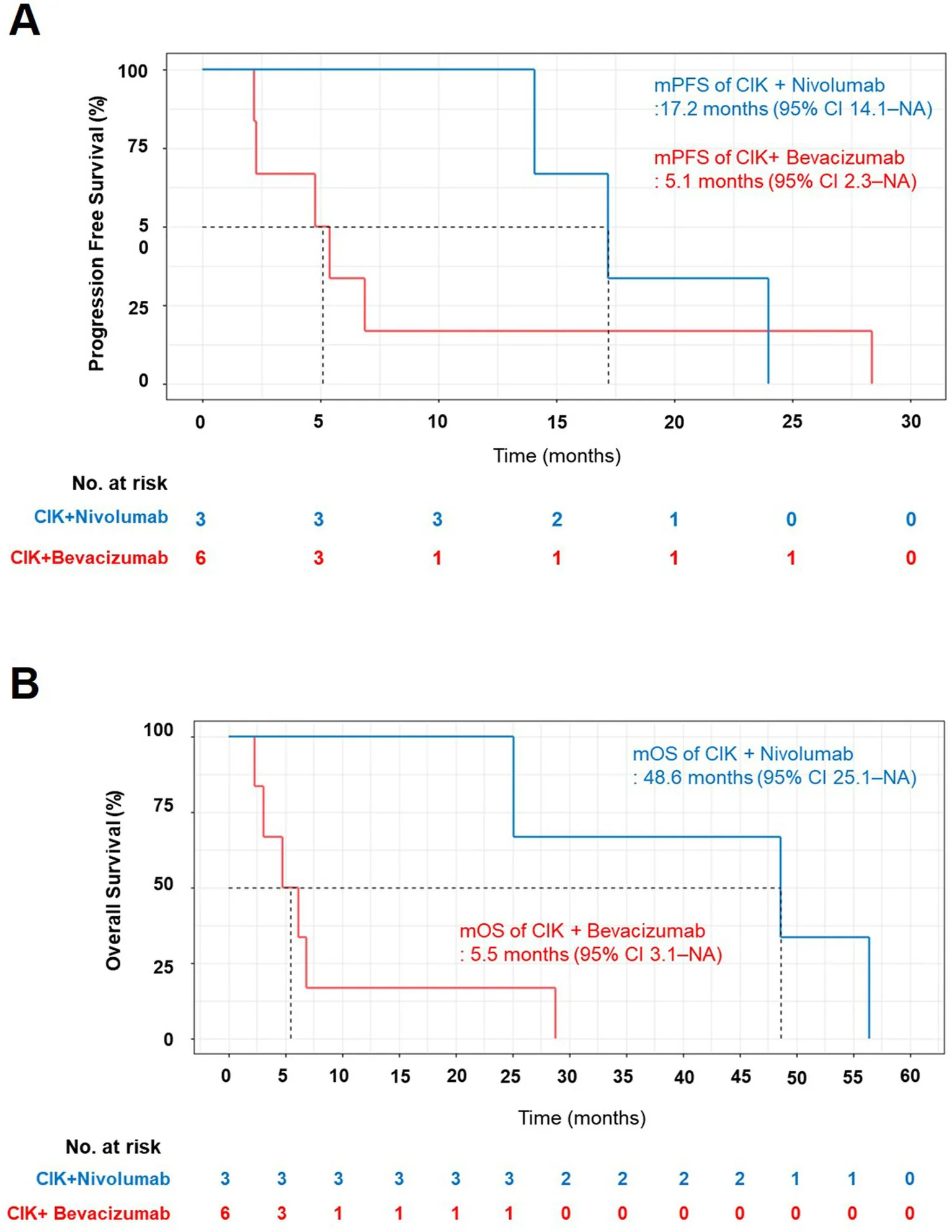
Exploratory survival analyses according to concomitant treatment regimen. Kaplan–Meier curves showing exploratory survival outcomes according to the concomitant treatment regimen administered with CIK-cell-based therapy. **(A)** Median PFS was 17.2 months (95% CI, 14.1–NA) in the CIK plus nivolumab subgroup (*n* = 3) and 5.1 months (95% CI, 2.3–NA) in the CIK plus bevacizumab subgroup (*n* = 6). **(B)** Median OS was 48.6 months (95% CI, 25.1–NA) in the CIK plus nivolumab subgroup and 5.5 months (95% CI, 3.1–NA) in the CIK plus bevacizumab subgroup. Numbers at risk are shown below each panel. Given the small subgroup sizes, retrospective study design, and non-randomized treatment allocation, these subgroup analyses are exploratory and descriptive and should not be interpreted as evidence of comparative treatment efficacy. NA, not available.

### Safety

CIK-cell-based therapy was generally well tolerated. Adverse events of any grade were reported in 83.3% of patients, and grade 3 or higher adverse events occurred in 25.0% of patients (Table 3). The most commonly reported adverse events included fatigue, myalgia, and anemia.

**Table 3:** Safety profile (*n* = 12).

Terms	Any grade No. of pts (%)	Grade 3/4 No. of pts (%)
Neutropenia	5 (41.6)	1 (8.3)
Anemia	6 (50.0)	3 (25.0)
Thrombocytopenia	3 (25.0)	1 (8.3)
Injection site reaction	2 (16.6)	0 (0.0)
Urticaria	3 (25.0)	0 (0.0)
Fatigue	10 (83.3)	3 (25.0)
Peripheral neuropathy	4 (33.3)	0 (0.0)
Myalgia	6 (50.0)	1 (8.3)
Nausea/Vomiting	4 (33.3)	1 (8.3)
Diarrhea	2 (16.6)	0 (0.0)
Constipation	5 (50.0)	2 (16.6)
Stomatitis	1 (8.3)	0 (0.0)
Cough	3 (25.0)	1 (8.3)
Pneumonitis	1 (8.3)	0 (0.0)
Elevated liver enzyme	2 (16.6)	0 (0.0)
Renal insufficiency	1 (8.3)	0 (0.0)
Hypothyroidism	2 (16.6)	1 (8.3)

One patient experienced grade 3 nausea and vomiting considered possibly or probably related to CIK-cell therapy; the symptoms resolved with oral medication. No patients discontinued treatment because of adverse events, and no treatment-related deaths were observed. No cytokine release syndrome, anaphylaxis, or severe immune-related adverse event was reported.

## Discussion

In this study, we integrated phenotypic characterization of patient-derived CIK-cell products, an orthotopic LN229-luciferase GBM model, and a retrospective recurrent-GBM cohort. Quantitative reanalysis of the preclinical study demonstrated treatment-associated differences in longitudinal BLI trajectories and quantitative histological differences among groups, while not establishing a statistically significant incremental benefit of adding CIK cells to TMZ. In the retrospective clinical cohort, CIK-cell-based therapy was feasible and generally well tolerated; however, the observed survival outcomes remain descriptive because of the small sample size, treatment heterogeneity, and absence of a comparable control group.

The expanded CIK-cell products were enriched for CD8-dominant effector-memory-like lymphocytes and expressed high levels of NKG2D and DNAM-1. NKG2D and DNAM-1 are activating receptors that can contribute to the NK-like, non-MHC-restricted tumor-recognition properties of CIK cells (7). NKG2D ligands, including MICA/B and ULBPs, have been detected in malignant glioma cells and specimens, while DNAM-1 ligands such as CD155/PVR and CD112/Nectin-2 have been demonstrated in patient-derived GBM cells (18, 19). Nevertheless, NKG2D- and DNAM-1-ligand expression was not directly assessed on the LN229 cells used here, and LN229-specific killing, cytokine-release, CD107a-degranulation, and receptor-blocking assays were not performed. Therefore, receptor expression in this study should be interpreted as an activation-associated phenotype potentially relevant to tumor recognition rather than direct functional evidence. Similarly, relatively low PD-1 expression and high TIM-3 expression do not establish a functionally non-exhausted or exhausted phenotype, respectively, in the absence of functional exhaustion assays.

The biological rationale for combining TMZ with adoptive cellular therapy extends beyond its direct cytotoxic effect. Preclinical studies have shown that TMZ-induced tumor-cell damage can modulate tumor immunogenicity and the local immune environment, and dose- and schedule-dependent TMZ-induced lymphodepletion can alter host immune homeostasis and enhance the expansion or activity of adoptively transferred T cells (20–22). These observations provide a biological rationale for combining TMZ-mediated tumor control and immune modulation with cellular therapy. However, the present study did not directly evaluate TMZ-induced immunogenic cell death, antigen presentation, lymphodepletion-mediated CIK-cell expansion, or other mechanisms of interaction between TMZ and CIK cells. Accordingly, these mechanisms are considered potential biological explanations rather than mechanisms established by the current data.

The longitudinal BLI reanalysis refined the interpretation of the orthotopic experiment (Figure 2). A significant treatment-by-time interaction was observed among the control, TMZ, and TMZ + CIK groups through Week 4, and both TMZ-containing groups showed lower BLI radiance than control at Week 4. However, TMZ and TMZ + CIK did not differ significantly at that time point, and the exploratory full-period comparison through Week 8 did not demonstrate a statistically significant treatment-by-time interaction. Thus, the data support treatment-associated antitumor activity of the TMZ-containing regimens relative to control but do not establish synergy or a statistically significant incremental benefit from CIK-cell addition. The tissue-level analyses, now presented separately in Figure 3, provide complementary but limited histological evidence. Quantitative H&E analysis showed an overall group difference in histologically detectable tumor involvement, but the analysis was based on a limited number of single sections and does not represent stereologic total tumor volume. Granzyme B and CD56 staining did not show significant group differences. In the absence of direct CIK-cell labeling, species-specific validation, and appropriate negative controls, these IHC findings cannot establish CIK-cell infiltration or CIK-specific cytotoxic activity.

The translational application of adoptive cellular therapy in GBM remains challenging because of the profoundly immunosuppressive tumor microenvironment, restricted and heterogeneous immune-cell trafficking across the blood–brain and blood-tumor barriers, T-cell dysfunction and exhaustion, and marked intratumoral heterogeneity (4, 5, 23–25). These barriers may limit both the magnitude and durability of antitumor immune responses. The nude-mouse xenograft model used here enables evaluation of human tumor cells and human CIK-cell treatment in the brain but cannot recapitulate an intact adaptive immune system or the full immune complexity of human GBM.

The clinical findings require particularly cautious interpretation. The cohort included only 12 heavily pretreated patients, concomitant regimens were heterogeneous, and no contemporaneous matched or randomized comparator group was available. Consequently, the observed median PFS and OS cannot be causally attributed to CIK-cell therapy. The exploratory nivolumab- and bevacizumab-containing subgroup analyses are likewise hypothesis-generating only and cannot establish comparative treatment effects. Although major prognostic variables were considered, reliable multivariable adjustment was not performed because the small cohort and limited number of events would create a high risk of model overfitting and unstable estimates. Detailed patient-level prior-treatment sequences and exact recurrence counts also could not be uniformly reconstructed from the retrospective records.

The safety findings were consistent with previous clinical experience with CIK-cell therapy in solid tumors (26–30). No treatment discontinuation due to adverse events, cytokine release syndrome, anaphylaxis, or treatment-related mortality was observed. These data support the feasibility and tolerability of CIK-cell-based therapy in this recurrent-GBM cohort but should not be interpreted as evidence of efficacy.

Several additional limitations should be noted. The animal experiment included only four mice per group and was not supported by a formal *a priori* power calculation, limiting statistical precision and generalizability. No CIK-alone arm was included, preventing assessment of the independent contribution of CIK cells. One control mouse died from tumor progression after Week 4, resulting in outcome-related missing longitudinal measurements that were not imputed. Formal investigator blinding was not prespecified or documented for the original BLI or qualitative histological assessments, although the additional H&E quantification was performed using randomized image identifiers before treatment-group unblinding. Histological and IHC analyses were limited by the number of available sections, absence of negative controls for the retrospective IHC analysis, lack of direct CIK-cell tracking, and absence of additional mechanistic and functional assays. The clinical component was retrospective, small, heterogeneous, lacked a control group and blinded independent central review, and was not suitable for reliable multivariable prognostic modeling.

Overall, the revised quantitative preclinical analyses demonstrate treatment-associated antitumor activity in the orthotopic LN229 model, while the retrospective clinical data support feasibility and tolerability of CIK-cell-based therapy in recurrent GBM. Neither a statistically significant incremental preclinical benefit of adding CIK cells to TMZ nor clinical therapeutic efficacy can be established from the present study. Prospective, adequately powered controlled studies evaluating TMZ–CIK combination therapy, together with mechanistic studies incorporating direct CIK-cell tracking and functional validation, are required to define its potential clinical role.

## Data Availability

The datasets generated and analyzed during the current study are available from the corresponding author upon reasonable request.
